# Incidental Detection of Adrenal Myelolipoma: A Case Report and Review of Literature

**DOI:** 10.1155/2013/789481

**Published:** 2013-02-20

**Authors:** Junaid Nabi, Danish Rafiq, Fatema N. Authoy, Ghulam Nabi Sofi

**Affiliations:** ^1^Department of Surgery, Shaheed Suhrawardy Medical College and Hospital, Dhaka 1207, Bangladesh; ^2^Department of Pathology, Sher-I-Kashmir Institute of Medical Sciences, Srinagar, Jammu and Kashmir, India

## Abstract

*Introduction*. Adrenal myelolipoma is a rare tumor that is benign in nature, usually asymptomatic, unilateral, and nonsecreting. It is composed of variable mixture of mature adipose tissue and hematopoietic elements and develops within the adrenal gland. With the widespread use of cross-sectional imaging modalities such as ultrasonography and computed tomography, the incidental detection of these tumors is increasing in frequency. *Case Presentation*. We report a case of adrenal myelolipoma in a 63-year-old Kashmiri male, who presented with pain in the right upper abdomen. Physical examination was unremarkable. Ultrasound abdomen showed the presence of a hyperechoic mass in the right suprarenal region with undefined margins. Contrast-enhanced computed tomography (CECT) scan of abdomen revealed a well-defined, round lesion in the right suprarenal region with heterogeneous attenuation suggesting the possibility of myelolipoma. The patient was subjected to right adrenalectomy and his postoperative course was uneventful. The histopathological evaluation of the mass confirmed the initial diagnosis of adrenal myelolipoma. *Conclusion*. Although mostly discovered as an “incidentaloma”, the diagnosis of adrenal myelolipoma warrants thorough diagnostic study. Imaging techniques such as ultrasonography and CT scans as well as biochemical studies are useful for indicating the best treatment taking into account the size of the mass and possible hormone production. Surgical resection is advocated through extraperitoneal approach as it minimizes postoperative complications and leads to quicker recovery.

## 1. Introduction

Adrenal myelolipoma is a rare urological lesion, benign in nature, and composed of variable mixture of mature adipose and hematopoietic elements. It was initially described by Gierke in 1905 and subsequently termed as *formations myelolipomatoses* by Oberling in 1929 [1]. In the past, these lesions used to be primarily detected at autopsy or in conditions where massive growth or an alteration in the hormonal production led to clinical presentation. However, in recent times, as a result of widespread use of noninvasive cross-sectional imaging modalities such as ultrasonography (US), computed tomography (CT) and magnetic resonance imaging (MRI), incidental detection is more common [2].

The tumor appears to affect men and women equally and most commonly found between the fifth and the seventh decade of life [2]. Accounting for 3–5% of all primary tumors of the adrenals, the true incidence of these tumors is not known, although it is thought to be 0.08%–0.4%, with increased incidence noted in the later decades of life [3]. The majority of these tumors are unilateral, small, and asymptomatic although some bilateral myelolipomas have been described [2]. They are generally nonsecreting in nature, and only one case of secreting myelolipoma has been reported so far [3]. These lesions are often smaller than 4 cm in diameter, and the largest reported in the literature was 31 × 24.5 × 11.5 cm and weighed 6 kg [3]. After surgical resection, these lesions tend to not recur.

Despite their benign biology, these lesions can be a cause of dilemma for a urologist; we describe a case of incidental diagnosis of adrenal myelolipoma in a patient who presented with upper abdominal pain and review the literature on its etiology, diagnosis, and management.

## 2. Case Presentation

**Figure 1:**
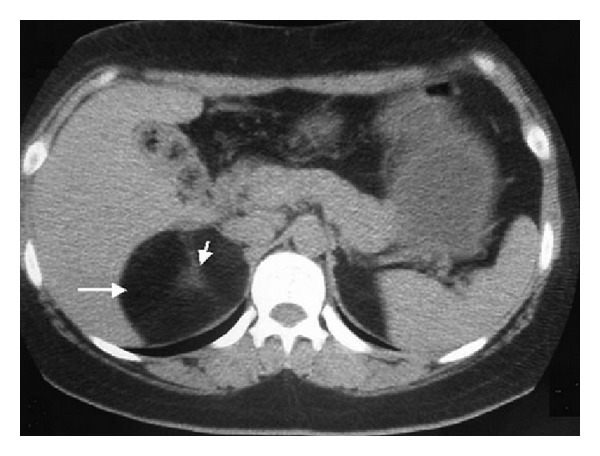
CT appearance of myelolipoma. Contrast-enhanced CT scan of the upper abdomen showing the heterogeneous mass covering upper right retroperitoneal space (arrows) with variable central and peripheral attenuation.

A 63-year-old Kashmiri male presented with the complaint of pain in the right upper abdomen for 16 days. The pain was colicky in character, of intermittent nature, and occasionally radiated to the back. On physical examination, there was no significant finding. Routine investigations such as hematological parameters were within normal limits. Ultrasonography (US) showed the presence of a hyperechoic mass with non-well-defined boundaries in the right suprarenal region measuring 5.9 × 4.5 cm. Computed tomography (CT) scan of the abdomen with a multidetector row CT (MDCT) was performed to evaluate the mass. Contrast-enhanced CT scan (CECT) revealed a well-defined, round lesion with central soft tissue attenuation (38–42 HU), and peripheral fat attenuation (−52 to −65 HU) measuring 6.1 × 4.0 cm was noted in the right suprarenal region (Figure 1). Location and attenuation of the mass on CT were suggestive of right adrenal myelolipoma.

**Figure 2:**
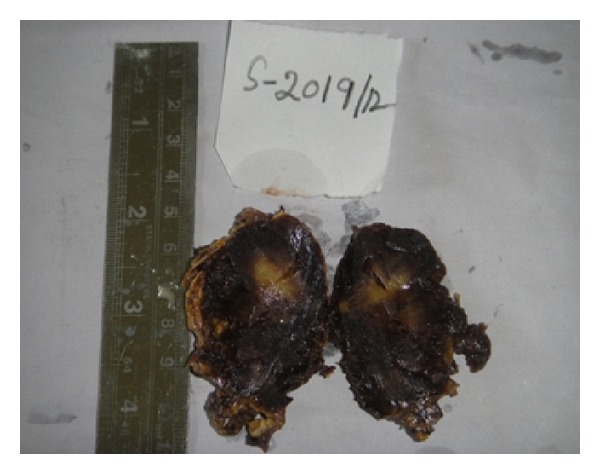
Cut surface of adrenal myelolipoma showing a variegated appearance of dark brown and yellowish areas.

**Figure 3:**
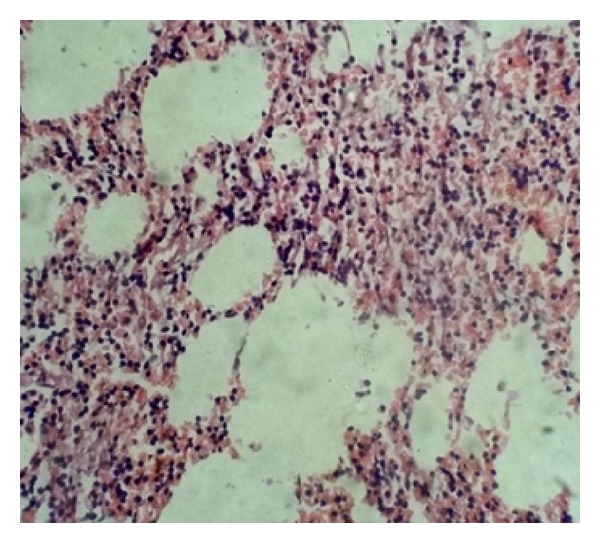
Microscopic appearance of adrenal myelolipoma. Typical histological features of myelolipoma comprising varying proportions of adipose tissue admixed with areas of hematopoietic tissue (H&E stain, ×40).

After a thorough preoperative workup, a surgical right adrenalectomy was performed through right subcostal incision for extraperitoneal approach of the adrenal gland. The mass was totally dissected from the upper pole of the right kidney, excised en bloc with the right adrenal gland, and sent for histopathological evaluation. Gross examination of the specimen revealed a large, rounded, and encapsulated mass with smooth external surface measuring 6.5 × 3.5 × 2.6 cm. Cut surface revealed a solid tumor with a variegated appearance of dark brown and yellowish areas (Figure 2). Microscopy revealed a characteristic admixture of mature adipose tissue with hematopoietic elements (Figure 3) without signs of cell atypia, thus confirming the initial diagnosis of adrenal myelolipoma.

The patient had an uneventful postoperative course and was discharged on the postoperative day 7. Three months after surgery, the patient was pain-free, and no recurrent mass was seen on ultrasonography. 

## 3. Discussion

Adrenal myelolipoma constitutes a rare entity in urological practice. They are composed of variable proportions of mature adipose tissue and active hematopoietic elements. They are also called “incidentalomas” since their diagnosis is based on autopsy or imaging modalities which are performed for reasons usually unrelated to adrenal diseases. Incidence ranges from 0.08% to 0.4%, and less than 300 cases were reported in the literature before 2000 [4]. However, their prevalence appears to be increasing up to 10%, due to the increased use of noninvasive and enhanced imaging techniques [5].

There are several theories for the etiology and the natural history of adrenal myelolipoma [6–8]. However, the most widely accepted theory is adrenocortical cell metaplasia in response to stimuli, such as necrosis, inflammation, infection, or stress [9]. This chronic stimulation to the adrenal gland, which is evidenced by the increased incidence of the lesion in the advanced age [10], could trigger the development of benign as well as malignant tumors. The conditions often associated with adrenal myelolipomas include Cushing's disease, obesity, hypertension, and diabetes which can be characterized as major adrenal stimuli [11]. Other contemporary authors have speculated about a stressful lifestyle and an unbalanced diet as factors that may be involved in the pathogenesis of this tumor [11]. Several case series have reported the predominance of the tumor in the right adrenal gland [12], which is yet to be explained. 

Ultrasonography, computed tomography, and MRI are all effective in diagnosing more than 90% of adrenal myelolipoma on the basis of identification of fat, with CT scan being the most sensitive [2, 12]. Since these tumors are nonfunctional, endocrinological evaluations may not be useful, although there is a report of a secreting myelolipoma causing hypertension [3]. The differential diagnosis should include renal angiomyolipoma, retroperitoneal lipoma, and liposarcoma [13]. 

Management of adrenal myelolipoma should be considered on individual basis. Small lesions, which are asymptomatic and measure less than 5 cm, should be monitored over a period of 1-2 years with imaging controls. [11]. It is suggested that symptomatic tumors or myelolipomas larger than 7 cm should be surgically excised [2], so as to prevent a urological emergency since there are reports of spontaneous rupture and hemorrhage of the mass presented with life-threatening cardiovascular shock [14]. In cases such as ours, extraperitoneal approach is preferable than midline incision as it leads to quicker recovery of the patient and lesser postoperative complications [11]. This approach, however, is not indicated for masses larger than 10 cm or in cases where there are adhesions and infiltration of the surrounding structures [15].

## 4. Conclusion

Adrenal myelolipomas are rare tumors, mostly of benign nature, and clinically silent. However, their “incidental” diagnosis should warrant careful diagnostic study to plan appropriate treatment. Imaging modalities such as ultrasonography and computed tomography can yield the diagnosis for the physician, as in our case, and can indicate the best treatment taking into account the size of the tumor. There is increasing number of myelolipomas reported with endocrine abnormalities which necessitate the use of thorough preoperative workup including biochemical studies. Smaller, asymptomatic myelolipomas can be observed expectantly with surgical resection reserved for larger or symptomatic lesions.
